# Modeling longitudinal oral health status and pneumonia risk: secondary data analyses of an integrated dental-medical cohort

**DOI:** 10.1186/s12903-023-03629-0

**Published:** 2023-12-01

**Authors:** Richard L. Berg, Ingrid Glurich, Aloksagar Panny, Frank A. Scannapieco, Jeffrey Miecznikowski, Jeffrey VanWormer, Amit Acharya

**Affiliations:** 1 Office of Research Computing and Analytics, Marshfield Clinic Research Institute, Marshfield, WI USA; 2 Cancer Care and Research Center, Marshfield Clinic Research Institute, Marshfield, WI USA; 3 Clinical Informatics, Methodist Health System, Dallas, TX USA; 4 Department of Oral Biology, School of Dental Medicine, University at Buffalo, Foster Hall, Buffalo, NY USA; 5 Department of Biostatistics School of Public Health and Health Professions, University at Buffalo, Kimball Tower, Buffalo, NY USA; 6 Center for Clinical Epidemiology & Population Health, Marshfield Clinic Research Institute, Marshfield, WI USA; 7 Advocate Aurora Health, 3075 Highland Parkway, Suite 600, Downers Grove, IL 60515 USA

**Keywords:** Oral health, Pneumonia, Propensity score, Risk assessment, Probabilistic models

## Abstract

**Background:**

Mounting evidence indicates potential associations between poor oral health status (OHS) and increased pneumonia risk. Relative pneumonia risk was assessed in the context of longitudinally documented OHS.

**Methods:**

Electronic medical/dental patient data captured from 2007 through 2019 were retrieved from the integrated health records of Marshfield Clinic Health Systems. Participant eligibility initiated with an assessment of OHS, stratified into the best, moderate, or worst OHS groups, with the additional criterion of ‘no pneumonia diagnosis in the past 90 days’. Pneumonia incidence was longitudinally monitored for up to 1 year from each qualifying dental visit. Models were assessed, with and without adjustment for prior pneumonia incidence, adjusted for smoking and subjected to confounding mitigation attributable to known pneumonia risk factors by applying propensity score analysis. Time-to-event analysis and proportional hazard modeling were applied to investigate relative pneumonia risk over time among the OHS groups.

**Results:**

Modeling identified associations between any incident pneumonia subtype and ‘number of missing teeth’ (*p* < 0.001) and ‘clinically assessed periodontal status’ (*p* < 0.01), which remained significant following adjustment for prior pneumonia incidence and smoking. The hazard ratio (HR) for ‘any incident pneumonia’ in the best OHS group for ‘number of missing teeth’ was 0.65, 95% confidence interval (CI) [0.54 − 0.79] (unadjusted) and 0.744, 95% CI [0.61 − 0.91] (adjusted). The HR for ‘any incident pneumonia’ in the best ‘clinically assessed periodontal status’ group was 0.72, 95% CI [0.58 − 0.90] (unadjusted) and 0.78, 95% CI [0.62 − 0.97] (adjusted).

**Conclusion/clinical relevance:**

Poor OHS increased pneumonia risk. Proactive attention of medical providers to patient OHS and health literacy surrounding oral-systemic disease association is vital, especially in high-risk populations.

## Introduction

A growing evidence base supports association between pneumonia risk and predisposing factors related to oral health status (OHS) [1–3]. Evidence from molecular studies suggests occurrence of shifts in relative representation of host microbiota in both the oral cavity and lungs during transition from oral health to disease states [3, 4]. Shifts in microbial profiles correlate with poor OHS stemming from behavioral risk factors including poor oral hygiene practices and low compliance with oral healthcare access [5]. Moreover, host response has both local and systemic collateral effects that may become chronic or contribute to pathogenic processes in the oral cavity and respiratory system [4].

Poor oral hygiene contributes to establishment chronic dysbiosis that promotes emergence of periodontal disease (PD) and establishment of altered oral microbiomes favoring periodontal pathogens [6]. Concomitantly, commensal flora that establish and maintain oral health are reduced [7]. Immune responses are ineffective in clearing periodontal pathogens and further contribute to tissue damage, thereby exacerbating local and systemic inflammation [7]. Altered microbiota may permit oral cavity colonization by potential respiratory pathogens, establishing a reservoir for opportunistic infection.

Notably, the healthy lung is not sterile, with upper and lower airways each exhibiting sparse, but distinct microbiomes [4, 8]. Because of anatomic proximity, the constituent pulmonary microbiome may reflect presence of microbiota translocated from the oral cavity [4]. When OH status is poor and/or PD is present, risk for pulmonary colonization by pathogenic microbes increases. Shifts in microbial content and diversity of upper and lower respiratory tract microbiome have been observed during pneumonia episodes, including changes in the relative representation of oral microbiota [4].

Increased risk for pneumonia is dynamic and may vary with the level and duration of sub-optimal OH. The study posited that history of poor OH status (OHS) was associated with risk for pneumonia incidence. By retrospectively interrogating clinical ‘big data’ of an integrated medical-dental health system captured by its integrated electronic health record (iEHR), this study sought to examine potential contributions of OHS and risk for incident pneumonia through time-to-event analysis and statistical risk modeling of longitudinal clinical OHS across a 12-year temporal window through systematic tracking of defined surrogate indicators of oral health. Pneumonia incidence and frequency was tracked and statistically modeled among subjects partitioned into ‘worst’, versus ‘moderate’ and ‘best’ OHS subgroups based on clinical history definable within delimited longitudinal time frames proximal to emergent pneumonia episodes.

## Methods

The study was reviewed and approved by the Marshfield Clinic Health System Institutional Review Board for secondary use of patient data in creating analytical data sets to support the study and approved for a waiver of informed consent.

### Source population

The source population consisted of individuals regularly receiving care at Marshfield Clinic Health System (MCHS), a large, healthcare system of over 50 medical and dental centers offering integrated care delivery to a predominantly rural region of central, northern, and western Wisconsin, and part of Michigan’s upper peninsula. MCHS offers dental care through affiliation with the Family Health Center of Marshfield (FHC-M) Dental Centers, a network of 10 strategically established, regional dental centers within its service area. An estimated 85% of patients receiving dental care through the 10 FHC-M dental clinics also seek healthcare from MCHS. Because the MCHS features an integrated medical-dental electronic health record (iEHR), holistic oversight of medical-dental healthcare delivery to the observational cohort is feasible. MCHS also operates Security Health Plan of Wisconsin, a health maintenance organization (SHP HMO) that served as an additional health data source for the current study.

### Cohort assembly

The analytical cohort included patients who were: 1) age ≥ 21 years, 2) received an oral health examination at an FHC-M dental center between 2007–2019, and 3) had reasonably complete capture of their medical care within MCHS data systems. This latter criterion included medically homed patients who receive primary medical care at MCHS medical centers documented by ≥ 2 ambulatory visits over the previous three years, or had an assigned MCHS primary care provider, were SHP HMO members, and/or residents of the Marshfield Epidemiologic Study Area [9, 10]. Data from 2020 were excluded due to potentially confounding factors secondary to the COVID-19 pandemic, including temporary suspension of dental care at FHC Dental Centers and very restricted ambulatory visits at MCHS Medical Centers. For any given participant, follow-up began when all eligibility criteria were met, and ended when an outcome or censoring event occurred, as defined further below.

### Oral health exposures

Identification of dental visits associated with oral health evaluations was facilitated via application of CDT codes including D0120: periodic examination; D0150 comprehensive evaluation and D0180 comprehensive periodontal evaluation. Five measures of OH status at such visits were extracted from the iEHR. Measures included:

1. ‘number of missing teeth’ (excluding third molars); attributable to advanced PD or tooth decay.
2. ‘percent of teeth with periodontal pocket depth (PPD) ≥ 5 mm’;
3. ‘clinically determined periodontal stage’ assigned by dental professionals reflecting the updated clinical periodontal assessment tool and PD classification system based on guidelines issued by the American Academy of Periodontology (AAP) Task Force [11]. Scoring of the extent of PD was based on a 5-point scale reflecting measures of incremental clinical severity applying the following framework:
  ***Stage 1***: Documentation of bleeding associated with brushing/flossing;
  ***Stage 2***: Gingivitis characterized by gingival redness associated with inflammatory activity;
  ***Stage 3***: Early periodontitis associated with sore, swollen gums, detection of deepening PPD and clinical attachment loss (CAL);
  ***Stage 4***: Moderate periodontitis, associated with early gum recession, loose teeth due to advancing CAL and further deepening of periodontal pockets;
  ***Stage 5***. Advanced Periodontitis: documented by extensive increases in severity reflected by PPD and CAL measures, presence of inflammation and BOP, and radiographic assessments of extent of bone loss;
4. percent of teeth with bleeding on probing (BOP) indicating gingival inflammation; and.
5. percent of teeth with restorations due to caries.

Analyses were conducted separately for each of the 5 key OH measures. Since the OH measures were ordinal, with generally skewed or peaked distributions, the measures were partitioned for analysis into ‘best’, ‘moderate’ and ‘worst’ OH groups. Group ranges were determined by observed patient numbers, with the assumption that most health system patients would have relatively good OH. Worst OH group designation among the cohort was arbitrarily assigned to the 20% exhibiting poorest OH based on review of data from clinical OH examinations evaluating the five key OH measures.

### Pneumonia documentation, validation and subtype classification

Documented pneumonia events/episodes were initially abstracted using International Classification of Diseases Clinical Modification (ICD-CM 9/10) codes captured in the iEHR for clinic patients meeting eligibility criteria including documentation of OH status in the iEHR preceding pneumonia incidence. Patients were evaluated for subsequent pneumonia events identified and validated by searches for pneumonia-specific ICD codes in the iEHR and supporting documentation offered by Current Procedural Terminology (CPT) or other coding relevant to pneumonia validation for up to 365 days after the index dental visit. Restricting this evaluation to 365 days was an attempt to limit analyses to a window of time where, given potential changes in oral health, measures from a single dental evaluation should have relevance. Using a full year had the advantage of always including a full range of seasons. Sensitivity analyses varying the period (e.g., to 3 years) showed similar results.

With the exception of ventilator-associated pneumonia, pneumonia ICD coding is not specific to pneumonia subtypes, but mainly classified using a non-subtype-specific code or codes specific to causal pathogens. A rule-based classification algorithm derived by our study team via informatics approaches applied to data from the same cohort analyzed in the current study defined pneumonia subtype classification for: community acquired (CAP), hospital acquired (HAP), health care acquired (HCAP), ventilator-acquired (VAP) or aspiration pneumonia (ASP) [12]. Classification was undertaken only on cases validated for pneumonia diagnosis. Subtype designation required ≥ 2 diagnostic codes entered by physicians or radiologic confirmation of the diagnosis [12]. Natural language processing (NLP) for identifying terminology validating or negating presence of pneumonia documented in free text of MCHS radiology notes was further developed to leverage non-structured pneumonia-associated terminology to confirm or negate true pneumonia cases [13]. Availability of these informatics tools supported conduct of time to event modeling for: 1) ‘any pneumonia’ and 2) the CAP subtype, observed with highest frequency (55%) during the study’s temporal window.

### Analytical approach

Time-to-event analysis explored the temporal association of comorbid oral/dental disease history within a defined temporal window preceding incident pneumonia. The temporal period initiated with identification of an index dental visit documented by evaluation of OH status (T_0_). Confirmation of no diagnosis in a 90-day window preceding the dental visit was required to rule out on-going pneumonia. From T_0_, the observational window extended over a 365-day period and ended at the earliest identification of either: 1) a pneumonia event; or 2) a censoring condition (e.g., 365 days without pneumonia, loss to follow-up, or death unrelated to pneumonia). OH-related data from the index dental visit and any subsequent visits within the observational window were combined in a time-weighted mean.

Patient groups defined by stratification of an OH measure likely also differ with respect to other health-related variables with potential for confounding. In order to reduce the risk of confounding and provide high-dimensional balance between the OH groups prior to analysis, propensity score matching was carried out. A propensity score was developed using logistic regression to create a predictive score for ‘worst’ OH, separately for each of the 5 key OH measures. Propensity score modeling targeted variables historically examined as potential risk factors for pneumonia based on literature review to identify potential risk factors. Historical risk variables identified included: age, gender, year of visit, chronic liver disease, heart failure, cerebrovascular disease, Type II diabetes mellitus, elevated HbA1c or glucose measure; chronic kidney disease; neoplastic disease and/or chemo-/or-radiotherapy: hypertension; elevated cholesterol/lipid measures; pharmaceutical exposure for control of hypertension, hyperglycemia or hyperlipidemia; and smoking history for a subset of the cohort for whom these data were available in the iEHR as structured data.

The logistic model included all individual covariates, with potential addition of important interaction terms through forward selection (*p* < 0.001 to enter the model). Those assigned to the ‘worst’ group were individually matched 1:1 by propensity score to patients in the ‘best’ and ‘moderate’ groups (arbitrary allowable caliper width 20% of the estimated propensity score standard deviation). Initial comparisons between matched OH groups regarding pneumonia incidence after the index dental visit were based on product-limit (Kaplan–Meier) estimators and log-rank tests. The primary analyses of pneumonia incidence were based on proportional hazards (PH) models [14]. These PH models included adjustment for the matching variables and were fit both with and without adjustment for prior history of pneumonia as a binary (yes/no) variable. Confidence limits for hazard ratios (HR) and tests of significance were based on robust covariance estimates to account for the matching. Modeling was carried out for the full cohort with any validated pneumonia, irrespective of subtype and separately for pneumonia classified as CAP applying a rule-based pneumonia subtype classification algorithm [12].

SAS was used for all statistical analyses. Test significance was assessed at level 0.05.

## Results

Collectively 68,863 unique individuals with 150,164 documented dental visits during the observational window were included in the analytical cohort. For each analysis, participants were assigned to one of three OHS subgroups. For example, with ‘number of missing teeth’ as the surrogate for determining OHS, subgroups were:

1. ‘Best’ = 0–1 missing teeth (*n* = 29,777 patients with 69,414 visits);
2. ‘Moderate’ = 2–7 missing teeth (*n* = 23,038 patients with 50,882 visits); and.
3. ‘Worst’ =  ≥ 8 missing teeth (*n* = 16,048 patients with 29,868 visits).

**Fig. 1:**
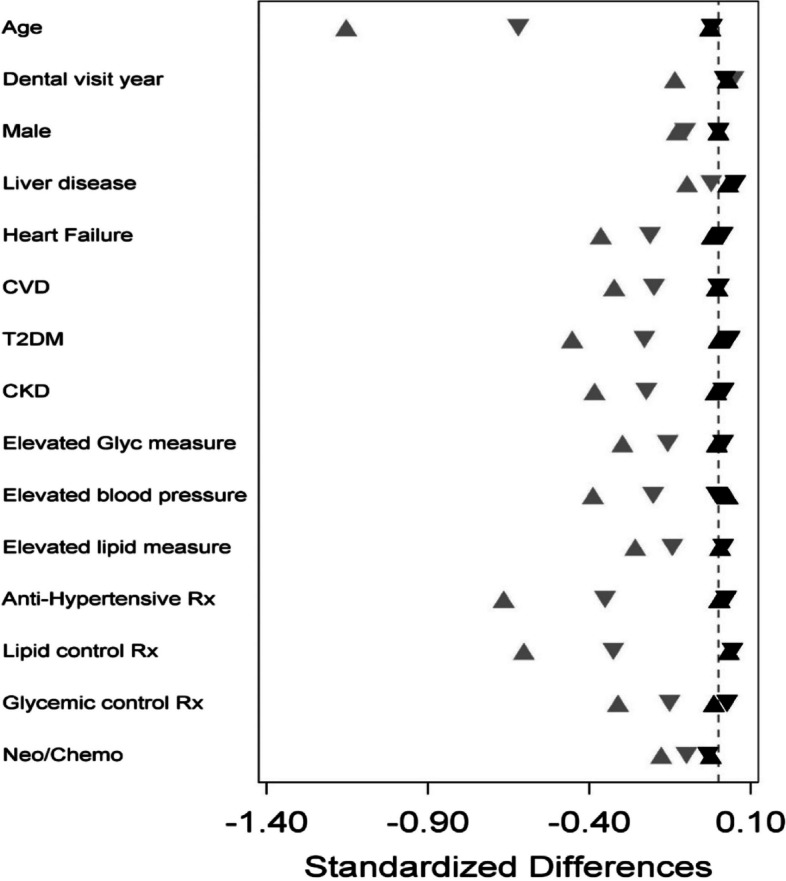
Propensity score adjustment to minimize potential confounding. Balance achieved with propensity score adjustment shows diminishing potential confounding by key variables associated with increased risk for pneumonia. Grey triangles indicate pre- adjustment and black triangles illustrate the matched groups. The dashed vertical line at 0 indicates the target of identical group means. Abbreviations: CVD = cardio/cerebrovascular disease; T2DM = Type 2 diabetes mellitus; CKD = chronic kidney disease; Glyc = glycemic; RX = prescription; neo/chemo = current neoplastic disease/chemo/radiation

Tempering of potential confounding contributed by other known risk factors for pneumonia was minimized by propensity score matching applying ‘number of missing teeth’ as the surrogate to define best, moderate and worst OHS. Figure 1 illustrates attenuation of confounding for variables included in propensity score analyses.

In addition to propensity score adjustments shown in Fig. 1, adjustment for smoking was applied to smoking data collected within the temporal window during which such data were electronically extractable (2010–2019) following the creation of fields in the iEHR to support electronic capture. Prior to 2010, such data were entered in the iEHR in a text-based format in medical notes and were not readily extractable electronically, and impact of earlier missing data was minimized by adjusting for smoking only in the subset of the study population for whom these data were electronically extractable. A statistically significant effect (*p* < 0.0001) for smoking status was observed (Table 1) suggesting high potential for confounding, which was minimized by adjusting for smoking in the PH models. Notably, the ‘worst OHS’ group exhibited the highest rates of current smokers and lowest rate of non-smokers, compared to the other two OHS groups.

**Table 1:** Smoking history and oral health status comparing patients grouped based on number of missing teeth (MT)

	**Smoking frequency (%)**				
***Missing Teeth (MT) Group Assignment***	***Current smoker***	***Former Smoker***	***Never smoker***	***Unknown Smoking status***	***Total***
***Best (*< *2 MT)***	**2,276** 23.2%	**2,638** 26.9%	**3,833** 39.1%	**1049** 10.7%	*9,796*
***Moderate (*≥ *2–7 MT)***	**3,251** 33.2%	**2,625** 26.8%	**2,969** 30.3%	**951** 9.7%	*9,796*
***Worst (*≥ *8 MT)***	**4,834** 49.3%	**2,304** 23.5%	**1,666** 17.0%	**992** 10.1%	*9,796*
***Total***	***10,361***	***7,567***	***8,468***	***2,992***	***29,388***
	***Statistic***	DF*	***Value***	***P value***	
	Chi** -**Square	6	1841.6	< 0.0001	

*DF = degrees of freedom

**Fig. 2:**
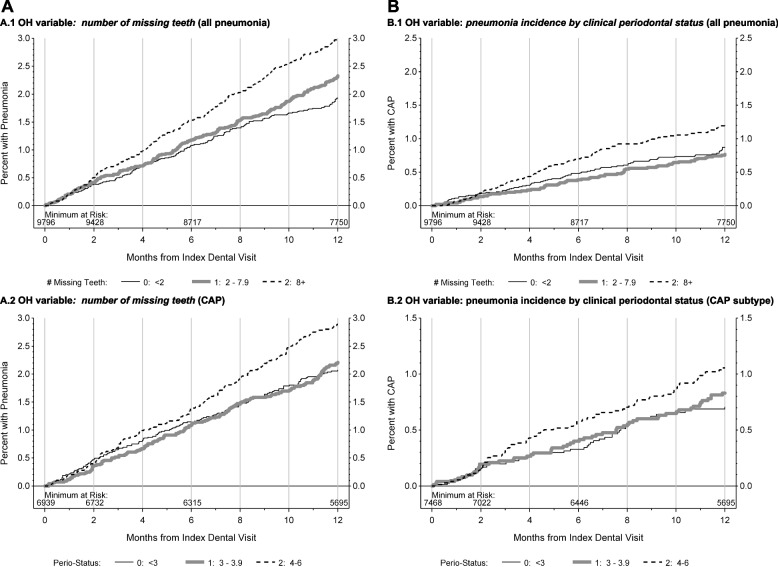
Kaplan Meier plots comparing Worst OH status vs. moderate or minimal oral disease subgroups for OH-related risk factors for any pneumonia (Fig 2A) and CAP (Fig 2B). Patients with worst OHS and highest number of missing teeth (>/= 8), (designated by large-dashed line), were compared with patients with moderate OHS and tooth loss (2-7 Teeth) (designated by small dashed line) and best OHS with low tooth loss (< 2) (designated by solid line) for the following oral health variables: A.1. Patients with >/= 8 missing teeth showed higher rates of any incident pneumonia compared to patients with 0 to 7 missing teeth over time (*p* < 0.0001). A.2. Patients with clinical PD ranking for severe periodontal disease (PD) compared to those ranked with moderate or low/no PD showed highest risk for emergence for incidence of any pneumonia over time (*p* = 0.0056). B.1. Patients with >/= 8 missing teeth showed higher rates of any incident CAP compared to patients with 0 to 7 missing teeth over time (*p* = 0.0004). B.2. Patients with clinical PD ranking for severe periodontal disease (PD) compared to those ranked with moderate or low/no PD showed highest risk for CAP emergence over time (*p* = 0.0144). No significant differences in bleeding on probing (BOP) and PPD ≥5mm were noted in comparisons across groups. Analyses also found no evidence supporting association of increased pneumonia risk for the worst OHS group for the variable: ‘% of teeth with dental caries/restorations’. (Data not shown)

Kaplan Meier analyses comparing’worst’ OHS subgroup to ‘moderate and best’ OHS subgroups carried out on the propensity score-adjusted matched cohort data set are shown in Fig. 2. Highest rates of ‘all pneumonia’ and ‘CAP’ incidence are noted among the ‘worst OHS subgroup’ strata. CAP pneumonia subtype represented 55% of all pneumonia following sub-classification of the same cohort modeled in the current study and was also analyzed independently [12]. Kaplan Meier analyses of ‘all pneumonia’ and ‘CAP’ showed statistically significant outcomes for: a) ‘number of missing teeth’ and b) ‘clinical periodontal status ≥ III’ (see Fig. 2).

Table 2 summarizes comparison of maximum standardized absolute differences for each of the targeted characteristics for each OHS subgroup defining both unadjusted (Table 2A) and adjusted values (Table 2B) following propensity matching. Greatly reduced absolute standardized differences were noted for the propensity score-matched cohort (Table 2B), demonstrating attenuation of potential confounding of group comparisons. The magnitude of standardized differences for each variable shown graphically in Fig. 1, illustrated improved balance achieved following matching (black plot symbols).

**Table 2:** Baseline characteristics of full cohort following 3-way stratification defined by # of missing teeth

**A: Unadjusted**				
**Variable measures within temporal observational window**	**Best**^a^** OH (*n* = 69,414 visits in 29,777 patients)**	**Moderate**^b^** OH (*n* = 50,882 visits in 23,038 patients)**	**Worst**^c^** OH (*n* = 29,868 visits in 16,048 patients)**	**Max. Absolute Standardized Difference**
**Age**	41.6 ± 14.8	49.3 ± 16.1	59.2 ± 15.8	1.153
**Dental Visit Year**	7.2 ± 3.5	7.8 ± 3.3	7.6 ± 3.3	0.135
**Male Gender**	25,285 (36.4%)	19,146 (37.6%)	12,749 (42.7%)	0.128
**Liver disease**	1976 (3.0%)	2151 (4.2%)	1400 (4.7%)	0.097
**Heart Failure**	1112 (1.6%)	2289 (4.5%)	2975 (10.0%)	0.364
**CVD**^d^	1905 (2.7%)	2717 (5.3%)	3207 (10.7%)	0.323
**T2DM**^e^	5820 (8.4%)	7982 (15.7%)	7416 (24.8%)	0.453
**CKD**^f^	2234 (3.2%)	3541 (7.0%)	4095 (13.7%)	0.384
**Elevated Glycemic measure**	2885 (4.2%)	3836 (7.5%)	3647 (12.2%)	0.297
**Elevated blood pressure**	12,189 (17.6%)	12,773 (25.1%)	10,239 (34.3%)	0.389
**Elevated lipid measure**	11,018 (15.9%)	10,317 (20.3%)	7853 (26.3%)	0.258
**Anti-Hypertensive RX**^g^	14,027 (20.2%)	16,945 (33.3%)	15,041 (50.4%)	0.665
**Lipid control RX**^g^	9711 (14.0%)	12,489 (24.5%)	11,809(39.5%)	0.603
**Glycemic control RX**^g^	3975 (5.7%)	5139 (10.1%)	4515 (15.1%)	0.311
**Neo/Chemo**^h^	5003 (7.2%)	4774 (9.4%)	3723 (12.5%)	0.177
**B: Baseline characteristics following propensity score adjustment**				
**Variable measures within temporal observational window**	**Best**^a^** OH (*n* = 9796 visits in 9796 patients)**	**Moderate**^b^** OH (*n* = 9796 visits in 9796 patients)**	**Worst**^c^** OH (*n* = 9796 visits in 9796 patients)**	**Max. Absolute Standardized Difference**
**Age**	49.2 ± 15.3	49.4 ± 15.3	49.6 ± 15.3	0.026
**Dental visit year**	7.0 ± 3.5	7.0 ± 3.4	6.9 ± 3.5	0.029
**Male gender**	4051 (41.3%)	3897 (39.7%)	4223 (43.1%)	0.000
**Liver disease**	342 (3.5%)	392 (4.0%)	306 (3.1%)	0.052
**Heart failure**	369 (3.8%)	405 (4.1%)	380 (3.9%)	0.019
**CVD**^d^	477 (4.9%)	477 (4.9%)	494 (5.0%)	0.004
**T2DM**^e^	1436 (14.6%)	1524 (15.5%)	1419 (14.5%)	0.035
**CKD**^f^	576 (5.9%)	605 (6.2%)	541 (5.5%)	0.016
**Elevated Glycemic measure**	684 (7.0%)	722 (7.4%)	718 (7.3%)	0.014
**Elevated blood pressure**	2816 (28.7%)	2609 (26.6%)	2709 (27.6%)	0.028
**Elevated lipid value**	1916 (19.5%)	2002 (20.4%)	1976 (20.2%)	0.015
**Anti-Hypertensive RX**^g^	3233 (33.0%)	3272 (33.4%)	3195 (32.6%)	0.024
**Lipid control RX**^g^	2322 (23.7%)	2375 (24.2%)	2266 (23.1%)	0.044
**Glycemic control RX**^g^	900 (9.2%)	988 (10.1%)	920 (9.4%)	0.027
**Neo/Chemo**^h^	854 (8.7%)	855 (8.7%)	892 (9.1%)	0.032

Oral health (OH) status definitions characterized by the most significant OH variable contributing pneumonia risk: ‘number of missing teeth’. This variable was stratified into 3 groups defined by incremental increase in # of missing teeth as follows: a) Best^a^: 0–1 missing teeth; b) Intermediate^b^: 2–7 missing teeth; Worst^c^: ≥ 8 missing teeth. Analysis is based on independent comparison of ‘Worst’ to: [‘Moderate’ and ‘Best’]. Continuous variables are reported as Mean (± SD); binary variables are reported as ‘N’ (%)

*Abbreviations*: ^d^CVD cardio/cerebrovascular disease, ^e^*T2DM* Type 2 diabetes mellitus, ^f^*CKD* chronic kidney disease, ^g^*RX* prescription, ^h^*neo/chemo* current neoplastic disease/chemo/radiation

Results of Cox proportional hazards (PH) modeling of OH variables on pneumonia risk are shown in Table 3, for any pneumonia, without adjustment for prior pneumonia and smoking status (3A) and following adjustment (3B). Modeling ‘worst’ versus ‘moderate’ and ‘best’ OHS subgroups unadjusted, and adjusted for prior pneumonia and smoking, identified ‘number of missing teeth’ and ‘clinical periodontal status ≥ III’ as having a strong and significant associations with risk for ‘any pneumonia’. *‘Number of missing teeth’* was the only OH variable that contributed significantly to CAP risk following Cox PH modeling involving comparison of ‘worst’ with ‘moderate’ and ‘best’ OHS subgroups both when unadjusted and following adjustment for prior pneumonia and smoking.

**Table 3:** Proportional Hazards Modeling OH variables for all pneumonia^*^

A. Without Adjustment for prior pneumonia or smoking history				
**Variable**	**Hazard Ratio**	**Lower limit**	**Upper limit**	**p-value**
**A.A. # of missing teeth**				
** Best vs Worst**	**0.65**	**0.54**	**0.79**	** < 0.001**
** Moderate vs Worst**	**0.77**	**0.64**	**0.92**	**0.005**
			3-group *p*-value	** < 0.001**
**A.B. % of teeth with PPD ≥ 5 mm**				
** Best vs Worst**	**0.93**	**0.80**	**1.08**	**0.335**
** Moderate vs Worst**	**0.79**	**0.68**	**0.93**	**0.005**
			3-group *p*-value	**0.017**
**A.C. Clinical Periodontal Status**				
** Best vs Worst**	**0.72**	**0.58**	**0.90**	**0.004**
** Moderate vs Worst**	**0.77**	**0.61**	**0.95**	**0.017**
			3-group *p*-value	**0.008**
**A.D. % of teeth with restorations**				
** Best vs Worst**	**1.21**	**1.01**	**1.44**	**0.038**
** Moderate vs Worst**	**1.04**	**0.87**	**1.25**	**0.652**
			3-group *p*-value	**0.087**
**A.E. % of teeth with BOP**				
** Best vs Worst**	**0.89**	**0.77**	**1.04**	**0.152**
** Moderate vs Worst**	**0.96**	**0.82**	**1.11**	**0.551**
			3-group *p*-value	**0.355**
**B. With Adjustment for pneumonia history and smoking history**				
**Variable**	**Hazard Ratio**	**(lower limit)**	**(upper limit)**	**p-value**
**B.A # of missing teeth**				
** Best vs Worst**	**0.74**	**0.61**	**0.91**	**0.003**
** Moderate vs Worst**	**0.83**	**0.68**	**0.99**	**0.043**
			3-group *p*-value	**0.010**
**B.B % of teeth with PPD ≥ 5 mm**				
** Best vs Worst**	**0.96**	**0.82**	**1.12**	**0.575**
** Moderate vs Worst**	**0.82**	**0.70**	**0.96**	**0.013**
			3-group *p*-value	**0.038**
**B.C. Clinical Periodontal Status**				
** Best vs Worst**	**0.78**	**0.62**	**0.97**	**0.029**
** Moderate vs Worst**	**0.82**	**0.66**	**1.02**	**0.074**
			3-group *p*-value	**0.061**
**B.D. % of teeth with restorations**				
** Best vs Worst**	**1.25**	**1.05**	**1.49**	**0.013**
** Moderate vs Worst**	**1.07**	**0.89**	**1.28**	**0.491**
			3-group *p*-value	**0.034**
**B.E. % of teeth with BOP**				
** Best vs Worst**	**0.89**	**0.76**	**1.03**	**0.118**
** Moderate vs Worst**	**0.97**	**0.84**	**1.13**	**0.715**
			3-group *p*-value	**0.262**
**Without adjustment for prior pneumonia or smoking history**				
**Variable**	**Hazard Ratio**	**Lower Limit**	**Upper Limit**	**p value**
**C.A # of missing teeth**				
** Best vs worst**	**0.71**	**0.53**	**0.95**	**0.021**
** Moderate vs worst**	**0.61**	**0.45**	**0.82**	**0.001**
			3-group *p* value	**0.003**
**C.B % of teeth with PPD ≥ 5 mm**				
** Best vs worst**	**0.91**	**0.72**	**1.16**	**0.464**
** Moderate vs worst**	**0.85**	**0.67**	**1.08**	**0.189**
			3-group *p* value	**0.418**
**C.C. Clinical Periodontal Status**				
** Best vs worst**	**0.68**	**0.47**	**1.00**	**0.048**
** Moderate vs worst**	**0.76**	**0.53**	**1.09**	**0.137**
			3-group *p* value	**0.109**
**C.D. % of teeth with restorations**				
** Best vs worst**	**1.10**	**0.83**	**1.46**	**0.506**
** Moderate vs worst**	**0.99**	**0.74**	**1.32**	**0.921**
			3-group *p* value	**0.702**
**C.E. % of teeth with BOP**				
** Best vs worst**	**0.74**	**0.58**	**0.93**	**0.011**
** Moderate vs worst**	**0.93**	**0.75**	**1.16**	**0.532**
			3-group *p* value	**0.033**
**D. With adjustment for prior pneumonia and smoking history**				
**Variable**	**Hazard Ratio**	**Upper Limit**	**Lower limit**	**p value**
3D.A # of missing teeth				
Best vs Worst	**0.81**	**0.60**	**1.10**	**0.177**
Moderate vs Worst	**0.65**	**0.48**	**0.89**	**0.007**
			3-group *p* value	**0.025**
**D.B % of teeth with PPD ≥ 5 mm**				
** Best vs Worst**	**0.96**	**0.76**	**1.22**	**0.730**
** Moderate vs Worst**	**0.88**	**0.69**	**1.13**	**0.317**
			3-group *p* value	**0.600**
**D.C. Clinical Periodontal Status**				
** Best vs Worst**	**0.72**	**0.49**	**1.06**	**0.097**
** Moderate vs Worst**	**0.80**	**0.55**	**1.15**	**0.225**
			3-group *p* value	**0.217**
**D.D. % of teeth with restorations**				
** Best vs Worst**	**1.15**	**0.86**	**1.52**	**0.346**
** Moderate vs Worst**	**1.02**	**0.76**	**1.36**	**0.889**
			3-group *p* value	**0.592**
**D.E. % of teeth with BOP**				
** Best vs Worst**	**0.74**	**0.59**	**0.94**	**0.012**
** Moderate vs Worst**	**0.96**	**0.78**	**1.19**	**0.701**
			3-group *p* value	**0.031**

Tables 3 summarizes key study outcomes derived by proportional hazards modeling of OH variables with potential for impacting on pneumonia risk for the following OH variables:

A. Number of missing teeth (MT);

B. Percent of teeth with periodontal pocket depth (PPD) ≥ 5 mm

C. AAP periodontal score (11) assigned during dental clinical assessment of periodontal parameters, where scores ≥ III, IV, V indicates PD of increasing severity, respectively;

D. Percent of evaluable teeth with restorations

E. Percent of teeth with bleeding on probing (BOP)

The following outcomes are summarized in Table 3:

(3A) Proportional hazard (PH) modeling outcomes of OH variables (A-E) relative to any incident pneumonia,

(3A.A-E): unadjusted for prior pneumonia;

(3B.A-E): adjusted for prior pneumonia and smoking history

(3C.A-E): PH modeling outcomes of OH variables for CAP subtype, unadjusted for prior pneumonia;

(3D.A-E): PH modeling outcomes of OH variables for CAP subtype adjusted for prior pneumonia and smoking history

PH modeling compared subgroups stratified based on good, intermediate and poor OHS groups for OH variables that contributed significantly to risk for any pneumonia examining both unadjusted data and data adjusted for prior pneumonia and smoking status. Significant *p* values for OH variables contributing to any pneumonia risk for unadjusted models included: a) *number of missing teeth* and b) *clinical periodontal status*. Following adjustment for prior pneumonia and smoking status, only *number of missing teeth* retained a significant *p* value

PH modeling of CAP pneumonia comparing groups stratified by good, intermediate and poor OHS identified *‘number of missing teeth’* as the OH variable that exhibited significant *p* values for CAP risk for both the unadjusted model, and model adjusted for prior pneumonia and smoking

## Discussion

The present study undertook secondary analysis of longitudinal clinical medical and dental ‘real world’, ‘big data’ captured longitudinally in an integrated healthcare delivery environment to: 1) examine association of OHS status and risk for: a) emergence of ‘any pneumonia’ or b) ‘CAP’; and 2) characterize OH measures associated with increased risk for ‘any pneumonia’ and ‘CAP’. Poor OHS indicated by >/= 8 missing teeth following modeling of good and moderate OHS versus poor OHS emerged as a significant risk factor for both ‘any pneumonia’ and ‘CAP’.

Our findings corroborate outcomes of two similar studies examining OHS in the context of pneumonia. Similar to our study, Son et al. (2020) applied Cox regression modeling to oral health assessment data from the national Korean dental cohort (*n* = 122,551) to evaluate relative contribution of OHS to pneumonia risk in 4681 emergent pneumonia cases [15]. As in current study, these authors applied time-to-event analyses and similarly found increased risk of incident pneumonia in association with ≥ 5missing teeth (*p* < 0.0001) [15]. Increased pneumonia risk associated with dental caries was also reported [15], but not found in the current study. Lower pneumonia rates among individuals reporting frequent tooth brushing or regular professional dental cleanings were reported by Son et al*.* [15], but not evaluated in the current study. Whereas the current study evaluated pneumonia risk for each qualifying OH status assessment longitudinally across a 12-year temporal window, Son et al. evaluated pneumonia risk based on OH status measured only in 2005–2006 while monitoring for any incident pneumonia through 2015 [15]. Thus, longitudinal changes in OH status over time were not assessed in their study.

Yang et al*.* (2020), examined the effect of oral disease treatment on pneumonia incidence in a Taiwanese population [16], an outcome not examined in the current study. Similar to the current study, Cox proportional regression analysis was applied across a longitudinal 12-year follow-up interval that parallels the observational window of the current study, in order to observe pneumonia incidence rates among subjects receiving longitudinal periodontal treatment compared with subjects from the general population with good OHS [16]. Following propensity score adjustment to mitigate confounding also performed in the current study, significantly lower pneumonia rates were reported (*p* < 0.001) among those receiving timely PD management compared with the dentally healthy population [16]. Although their study examined an endpoint not examined in the current study, application of similar statistical modeling similarly corroborated the conclusion that poor OHS is associated with increased pneumonia risk, since attenuation of PD severity following treatment was associated with statistically significant lower rates of pneumonia in their study [16].

In contrast to the statistical modeling undertaken in our study, Hata et al*.* (2020) undertook molecular analysis to compare presence of obligate anaerobic bacteria, including periodontal pathogens, in bronchoscopic fluid and sputum of patients with pneumonia, who simultaneously underwent oral hygiene evaluations. Anaerobic bacteria were found in 80% of lavage specimens, with significantly higher levels detected in subjects with poorest oral hygiene evaluation outcomes (*p* = 0.008) [17]. These data support the oral cavity as a likely source of the anaerobic bacteria present in lavage fluid from patients with poor oral hygiene evaluation scores. Presence of oral anaerobes in both sputum and bronchial lavage fluid samples further reinforced poor OH as a risk factor for pneumonia [17]. Applying a statistical approach, the current study further corroborates the physiological observations of Hata et al. in confirming presence of oral anaerobes in lavage fluids from the lungs of patients with pneumonia who were documented to have poor oral health status.

### Study strengths

The present study has notable strengths. The study sample size was large (e.g. 9,796 unique patients in each of three matched groups in Table 2B) due to availability of rich, ‘real-world’, clinical, ‘big data’ captured in the iEHR and enterprise data warehouse of MCHS for their large, well-defined population-based cohort. These data supported comparisons between the full MCHS patient population accessing medical and/or dental healthcare, including patients with and without prior pneumonia incidence over a 12-year temporal window. Notably, the FHC-M dental clinic network represents a regional a dental safety net. Thus, available data is representative of a broad patient sample spanning diverse economic backgrounds with dental access provision irrespective of third-party coverage or income status.

All incident pneumonia cases analyzed were subtyped applying rule-based classification algorithms [12] and validated through radiographic confirmation and [13], mitigating classification error. Propensity score matching, while reducing the full sample size for analysis, appeared to successfully abate confounding from other established potential pneumonia risk factors, supporting discernment of the true relative risk contributed by the OHS to pneumonia emergence. To further reduce confounding potentially associated with smoking history and pneumonia recurrence, data were adjusted when proportional hazard (PH) models revealed imbalances across our OH subgroups. Adjustment was possible only for electronically extractable smoking data in structured data fields in the iEHR and more limited for early years when data were captured in clinical notes. Higher rates of current smokers and lower rates of non-smokers were noted among individuals stratified to the poor OHS group when compared to those assigned to good or intermediate OHS groups, and were statistically significant. Smoking status adjustment relative to OH status tended towards attenuation, marginally reducing the estimated OH risk.

No other OH variables examined, including dental caries/restorations emerged as risk factors for pneumonia. Notably, dental caries have been less frequently related to systemic health outcomes, including pneumonia, in other analytical studies [18]. However, a recent systematic review by Broers et al. (2022) identified caries as a major underlying factor contributing to tooth loss and estimated that 36–53% of tooth extractions may be attributable to underlying caries while 24 to 38% of tooth loss was associated with periodontal disease [19].

### Study limitations

Some study limitations require acknowledgement. A selection bias is possible because the cohort only included patients with OH data documented by an FHC-M dentist. However, 75% of FHC dental center patients also sought medical care at our integrated healthcare system. Since cohort eligibility required availability of both medical and dental data, selection bias risk was mitigated by eligible cohort definition. Prior pneumonia history may be unknown if patients sought care outside of MCHS prior to becoming a MCHS patient, raising potential for classification error. However, multiple episodes of pneumonia were frequently documented for individual cohort members during the observational window, thereby supporting time-to-event analysis adjusted for prior pneumonia events. Initial OH status in combination with time-weighted analysis of additional documented OH examinations within the observational window, made OH status knowable for up to a year preceding a pneumonia event.

Interpreting OHS surrounding history of tooth loss predating the temporal window may be unknowable. However, number of missing teeth and clinical PD status documenting presence of severe PD emerged consistently as risk factors for any pneumonia when not adjusted for prior pneumonia. Taken together, these data support high rates of tooth loss as a surrogate for poor OHS over the lifespan.

Although propensity score matching was used to reduce potential confounding contributed by 15 pneumonia risk factors (Fig. 1), currently unknown factors may exist that could contribute to confounding among OH groups. Another potential limitation to consider is inaccuracy of data entry in the iEHR. However, to the greatest extent possible, algorithms developed to support this study [12, 13] were applied for pneumonia validation and sub-classification, required vetting of multiple levels of evidence in the iEHR to support pneumonia validation and sub-classification, while tooth level data were electronically extractable for all dental variables, with the exception of CAL which was frequently entered in clinical notes. Any records that were not classifiable were excluded from analyses. Manual validation of algorithm performance also found high accuracy for the classification tools developed to support this study [12, 13], supporting that data sets were well vetted for accuracy in determining presence of pneumonia, pneumonia sub-classification and dental variables modeled to stratify pneumonia risk across the three OHS strata.

Recently updated guidelines for PD assessment published by Tonetti et al*.* in 2018, recommend use of clinical attachment loss (CAL) as a key clinical variable [20]. However, since this study represents secondary analysis of data collected during a retrospective observational window, guidelines operative during the temporal observational window of this study (AAP consensus guidelines and 2014 updates [11]) were applied. While CAL was one of several components considered for clinical staging of PD, capture of CAL in dental health records throughout most of the observational window was more commonly entered in clinical notes, precluding electronic retrieval. Thus, independent evaluation of CAL held bias potential associated with large amounts of missing data since manual abstraction from thousands of records over 12 years was cost prohibitive. However, since CAL was available to dental practitioners, CAL would have been reflected in the composite clinical periodontal staging score which considers multiple variables. Notably, higher clinical periodontal staging scores were significantly associated with increased pneumonia risk. Thus, likelihood that independent evaluation of CAL would alter modeling outcomes is low.

### Conclusion and clinical relevance

The OH variable most associated with pneumonia was tooth loss largely attributable to infectious processes involving advanced destructive dental caries and PD as the most common causes for tooth loss [19]. As PD advances in severity, eventual tooth loss is attributed to complex interactions of the oral microbiome and host response that drives chronic inflammation, and destruction of periodontal tissue, the root and underlying bone supporting the tooth as the tooth loses its supportive infrastructure. In the context of dental caries, bacterial metabolites erode and destroy the enamel exposing the pulp and if untreated, the tooth cannot be repaired. In the current study, clinical periodontal status showed relatively strong associations with pneumonia incidence whereas percent of teeth with restorations did not. However, our study group posits that the presence of restorations also indicates that the patient had accessed dental care to arrest the destructive processes, potentially confounding examination of the true impact of untreated dental caries on tooth loss. Therefore, the impact of dental caries on tooth loss remains equivocal in the literature.

Notably, our dental centers represent a dental safety net that serves a largely rural disparity population with high representation of Medicaid patients who meet definitions of federal poverty levels, are uninsured, elderly, include ethnic/racial minorities who have had historically low access to dental care across their lifetimes, contributing to higher rates of poor OHS in this population. In the current study, number of missing teeth lost likely consequential to underlying infectious processes emerges as a representative surrogate of poor OHS and long-term OH neglect with high clinical relevance, especially in the context of oral-systemic disease interactions, including increased risk for pneumonia for individuals with a history of poor OHS.

Significant difference in rates of emergent pneumonia were consistently noted among individuals meeting classification criteria stratifying them to the ‘worst’ oral health group. Notably, older age was also associated with the ‘worst’ OH group. Age and poor dental health, risk factors identified for CAP in the systematic review by Almirall et al.(2017) [21], were also documented risk factors in the present study.

Pneumonia risk reduction among high-risk and aging populations with low OH literacy reflected gaps in understanding of the following: 1) gaps in knowledge concerning oral cavity care; 2) patient awareness of systemic health risks associated with poor OH, and why promotion and achievement of integrated health care delivery are important; 3) assurance of patient access to dental care in compliance with the periodicity recommended by the dental provider. Gaps in knowledge regarding any of these are potentially indicative of low oral-systemic health literacy and require interventional education, and in some cases, triage to appropriate providers to get health issues addressed. Notably, a survey conducted to examine knowledgeability and practice behaviors surrounding oral health assessment among medical primary care providers documented inadequate attention to patients’ OH [22]. Providers further reported lack of training and competency in making OH assessments [22].

With mounting evidence linking poor OH as a risk factor for pneumonia emergence, proactive attentiveness among medical providers to OH status of their patients is vital. Recognizing OH as a modifiable risk factor and educating patients regarding importance of engaging good oral hygiene is important especially among vulnerable patient populations experiencing dental care access barriers who are not receiving this education or preventive care from dental professionals. Proactive establishment of integrated medical/dental delivery featuring interdisciplinary communication, effective referral systems, and access to dental safety net operations to improve dental care access for vulnerable patients with no dental access will increase the probability of achieving better OHS across their lifetime, Finally, dental and medical professionals require continuing education on evidence-based oral-systemic health associations to ensure that patients receive appropriate interdisciplinary care and education.

## Data Availability

The datasets generated and/or analyzed during the current study are not publicly available due to their proprietary nature (clinical data inclusive of direct and indirect identifiers) but are available from the corresponding author on reasonable request.

## Abbreviations

CAL: Clinical attachment loss
OH: Oral health
OHS: Oral health status
PD: Periodontal disease
iEHR: Integrated medical-dental electronic health record
MCHS: Marshfield Clinic Health System
FHC-M: Family Health Center of Marshfield, Inc
SHP HMO: Security Health Plan of Wisconsin health maintenance organization
PPD: Periodontal pocket depth
AAP: American Academy of Periodontology
BOP: Bleeding on probing
ICD-CM 9/10: International Classification of Diseases Clinical Modification codes
CPT: Current Procedural Terminology codes
CAP: Community acquired pneumonia
HAP: Hospital acquired pneumonia
HCAP: Health care acquired pneumonia
VAP: Ventilator-acquired pneumonia
ASP: Aspiration pneumonia
NLP: Natural language processing
PH: Proportional hazard ratio
HR: Hazard ratio
